# Perceived Efficacy, Reduced Prescription Drug Use, and Minimal Side Effects of Cannabis in Patients with Chronic Orthopedic Pain

**DOI:** 10.1089/can.2021.0088

**Published:** 2022-12-05

**Authors:** Ari Greis, Eric Larsen, Conan Liu, Bryan Renslo, Anjithaa Radakrishnan, Adrianne R. Wilson-Poe

**Affiliations:** ^1^ Department of Physical Medicine and Rehabilitation, Rothman Orthopedic Institute at Thomas Jefferson University, Philadelphia, Pennsylvania, USA.; ^2^ Sidney Kimmel Medical College at Thomas Jefferson University, Philadelphia, Pennsylvania, USA.; ^3^ Dow Neurobiology, Legacy Research Institute, Portland, Oregon, USA.

**Keywords:** orthopedic pain, chronic pain, cannabis, opioids, MME, benzodiazepines

## Abstract

**Introduction:**

Although cannabis is widely used for the treatment of chronic pain, most research relies on patient self-report and few studies have objectively quantified its efficacy and side effects. Extant inventories for measuring cannabis use were not designed to capture the medically relevant features of cannabis use, but rather were designed to detect problematic use or cannabis use disorder. Thus, we sought to capture the medically relevant features of cannabis use in a population of patients with orthopedic pain and pair these data with objective measures of pain and prescription drug use.

**Materials and Methods:**

In this prospective observational study, orthopedic pain patients were enrolled in Pennsylvania's medical cannabis program by their treating pain management physician, received cannabis education from their physician at the time of certification, and purchased products from state-licensed cannabis retailers.

**Results:**

Medical cannabis use was associated with clinical improvements in pain, function, and quality of life with reductions in prescription drug use; 73% either ceased or decreased opioid consumption and 31% discontinued benzodiazepines. Importantly, 52% of patients did not experience intoxication as a side effect of cannabis therapy. Significant clinical benefits of cannabis occurred within 3 months of initiating cannabis therapy and plateaued at the subsequent follow-ups.

**Conclusions:**

This work provides a direct relationship between the initiation of cannabis therapy and objectively fewer opioid and benzodiazepine prescriptions. Our work also identifies specific subpopulations of patients for whom cannabis may be most efficacious in reducing opioid consumption, and it highlights the importance of both physician involvement and patient self-titration in symptom management with cannabis.

## Introduction

Despite their questionable efficacy for chronic pain,^1^ prescription opioid analgesics have been a mainstay in clinical pain management over the last three decades. The severity and scale of the North American opioid overdose epidemic has highlighted the genuine risks of physical dependence and overdose with prescription opioids.^2,3^ These risks have been exacerbated by the coronavirus disease 2019 (COVID-19) pandemic, during which opioid overdose deaths significantly increased.^4^ Because opioids are also associated with other pathological risks such as cognitive impairment and endocrinopathy,^5–7^ there is strong rationale to identify less harmful, yet efficacious, treatments for chronic pain.

Cannabis contains hundreds of biologically active molecules that modulate mammalian pain physiology. These include the canonical cannabinoid receptors (CB1, CB2), TRPV1, and PPAR receptors.^8^ Exhaustive reviews of the literature support the relative safety and analgesic efficacy of cannabis for chronic pain in adults,^9^ and the majority of patients registered in U.S. state-level medical cannabis programs report that chronic pain management is their primary reason for using cannabis.^10^

Cannabinoids and opioids interact in a number of ways. Pre-clinical and human studies have demonstrated analgesic synergy between these molecules.^11,12^ These findings suggest that cannabis could prevent dose escalation or facilitate dose reduction for individuals using opioids. Several clinical studies have demonstrated the opioid-sparing properties of cannabis with patients reducing their opioid consumption by ∼50% when given access to cannabis.^10,13–15^ However, population-level studies are somewhat conflicting. Some show that the legalization of cannabis has been associated with reduced opioid-related mortality, hospitalizations, and prescription,^16–18^ whereas other studies suggest a positive relationship between cannabis laws and opioid mortality.^19^

Previous studies largely rely on patient self-report and epidemiological analyses.^15,18,20^ They have been unable to correlate medical efficacy with cannabis use patterns or address the concern that psychoactive side effects of cannabis may outweigh any potential therapeutic benefits. The goal of this study was to capture the medically relevant features of cannabis use in orthopedic noncancer pain patients and to objectively quantify the efficacy of cannabis for pain management and prescription medication reduction. Primary outcomes included change in pain, physical health, and mental health scores. Secondary outcomes measures included change in opioid and benzodiazepine use. We hypothesized that the introduction of cannabis would be correlated with decreased pain, improved physical and mental health, and decreased prescription drug use.

## Methods

### Participants

Study participants (*N*=468) were patients at the Rothman Orthopaedic Institute, a large orthopedic practice in Philadelphia, PA. Patients with chronic pain were referred to a physical medicine and rehabilitation physician who specializes in pain management. Chronic pain diagnoses were based on the referring provider's discretion and included chronic low back pain, multifaceted pain (e.g., fibromyalgia and neuropathies), neck pain, and joint pain. The initiation of this study coincided with the implementation of Pennsylvania's medical cannabis program.Therefore, data regarding patients' previous experience with cannabis were not collected, and the study was considered prospective. Based on the prevalence of cannabis use in states where cannabis is illegal, up to 7.4% of participants may have been daily cannabis users before enrolling in the current study.^21,22^ Medical records were reviewed and screened for history of mental health disease and substance abuse. The Pennsylvania Drug Monitoring Program (PDMP) website was queried for recent controlled substance use. Cannabis active ingredients and routes of delivery were reviewed with patients by the physician, and patients were provided guidelines on product selection. The detailed patient handout form regarding cannabis counseling can be found in the Supplementary Data. Patient applications for cannabis treatment were certified by a Rothman physician and sent to the Pennsylvania Department of Health for approval. All participants read and signed the consent form on the potential risks and benefits of the intervention.

### Procedure

Patient outcome measures were obtained by querying the Rothman Orthopaedic Cannabis Data Repository (ROCDR). The establishment of the data repository was approved by the Institutional Review Board of Thomas Jefferson University, Philadelphia, PA (protocol #19D.159). Prospective data collection took place between March 1, 2018, and January 31, 2020. Any participant who completed at least one follow-up within 12 months of certification was included in the analysis. One hundred four individuals who were certified to participate in PA's medical cannabis program but failed to follow up were not included in the analysis. Participants self-selected cannabis products and purchased them from state-licensed retailers.

### Measures

Prospective measures were queried at 3, 6, and 12 months following the initiation of cannabis therapy. The data analysts querying patient outcomes were not involved in participant recruitment or patient care. The analgesic efficacy of cannabis was assessed with the Visual Analog Scale (VAS) and the Patient-Reported Outcomes Measurement Information System; Global Physical Health and Global Mental Health (PROMIS). Quality of life (QoL) was quantified with the EuroQoL EQ-5D. Patterns, frequency, perceived efficacy, and side effects of cannabis use were measured using a purpose-built survey, the Inventory of Medical Cannabis Use (iMCU).

Prescription drug use was analyzed by accessing PDMP and collecting prescription data for a 6-month period before, and 6-month period following enrollment in PA's medical cannabis program. Total MME per patient was calculated by multiplying PDMP-provided raw daily MME by the number of days the patient had an active opioid prescription. Daily opioid consumption was normalized by dividing each patient's total MME prescribed in a 6-month period by the number of days that the patient had an active opioid supply.

The primary outcomes were pain reduction and general well-being improvement after 3, 6, and 12 months of cannabis therapy. Secondary outcomes included a change from baseline in the following: (1) prescription drug use (PDMP), (2) presence of side effects (iMCU), and (3) patient-reported cannabis analgesia (iMCU).

### Statistics

Survey data were analyzed using GraphPad Prism, version 9. A change from baseline of primary and secondary outcomes was assessed by the Wilcoxon signed rank test for non-normally distributed data. The normality of data distribution was assessed by the Shapiro–Wilk test. Repeated-measures analyses were conducted using mixed-effects analysis with Tukey's multiple comparisons test. Statistical significance level was set at *p*<0.05. Statistics was based on a total of 468 patients who completed baseline assessments at the time of certification, and had at least one follow-up within 12 months. Eighty-three patients failed to follow-up at 3 months, but followed up at a later time point. Two hundred twenty-six patients followed up at the 6-month time point, and 157 followed up the 12-month time point.

## Results

### Demographics

The average participant age was 61.1 years, and 56% of the participants were female (205 males, 263 females). The most prevalent reasons that participants were seeking treatment at the clinic were chronic low back (*N*=257) and multifaceted pain (e.g., fibromyalgia and neuropathies, *N*=111), followed by neck (*N*=63) and joint pain (shoulder, hip, knee, *N*=37).

### Cannabis use patterns

**FIG. 1.**
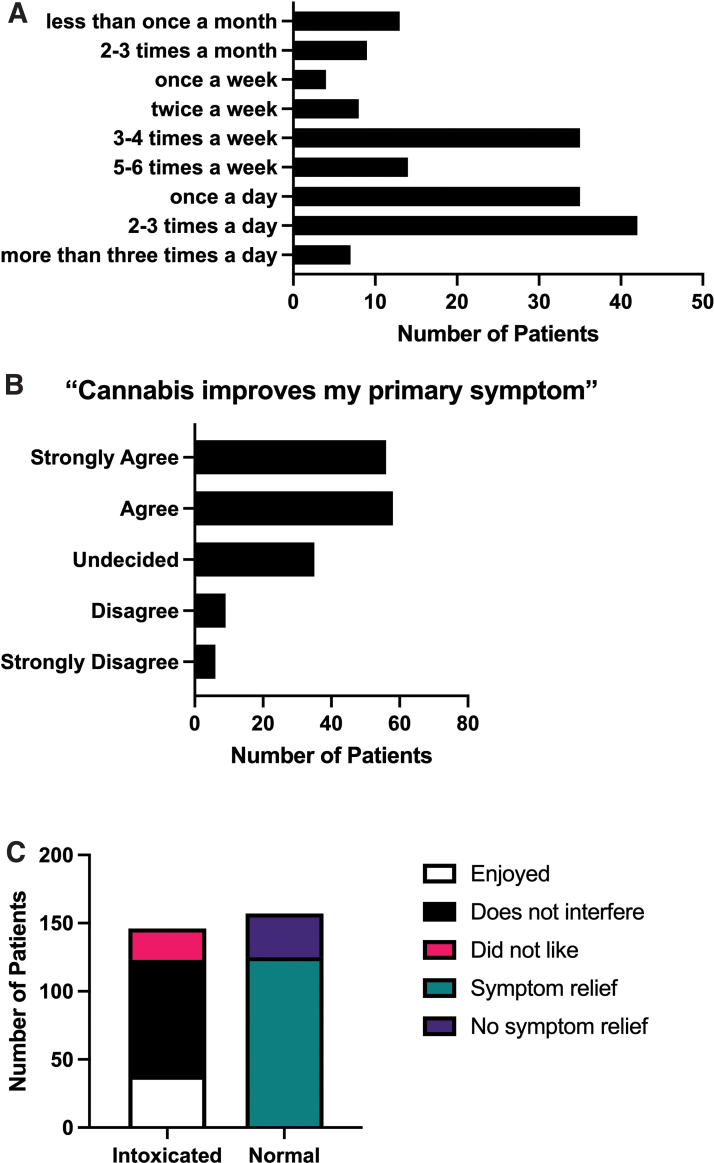
Patterns of medical cannabis use in a subset (*N*=328) of patients in this study. **(A)** Self-reported frequency of medical cannabis use: 25% of respondents reported using cannabis two to three times per day; once per day=21%; five to six times per week=8%; three to four times per week=21%. **(B)** Self-reported efficacy of medical cannabis use: Seventy percent of patients either agreed or strongly agreed that cannabis alleviated their primary symptom of pain. **(C)** Fifty-two percent (*N*=157) of patients reported that they did not experience intoxication. The most commonly reported experience of the patients was relief of pain in the absence of intoxication (*N*=125, 41%). Of the patients who did experience intoxication (*N*=146), 84% either found the intoxication to be enjoyable, or that it did not interfere with daily tasks. Color images are available online.

A subset of the total participants (*N*=328) completed all or part of the iMCU. Fifty percent of the participants who completed the iMCU used cannabis at least once daily, and 70% either agreed or strongly agreed that cannabis alleviated their primary symptom of pain (Fig. 1A, B). The most commonly reported frequency of cannabis use was two to three times per day, followed by an equal number of patients who either used once per day or three to four times per week. Although participants were asked to report the THC content of their cannabis preparation, only 23% were able to estimate the THC content of their inhalation product, and 29% were able to estimate the THC content of their oral product. When asked whether they experienced intoxication, 52% (*N*=157) of patients reported that they did not (Fig. 1C). The most commonly reported experience of the patients was relief of pain in the absence of intoxication (*N*=125, 41% of all patients). Of the patients who did experience intoxication (*N*=146), 84% either found the intoxication to be enjoyable, or that it did not interfere with daily tasks.

**FIG. 2.**
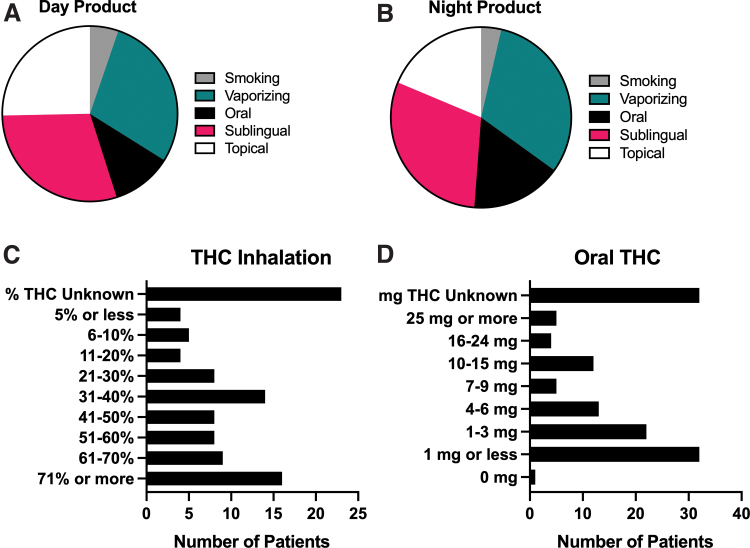
Self-reported route of administration and cannabinoid content of cannabis products used for pain relief. **(A)** Of *N*=304 patients who responded to questions regarding day-time use, 29% (*N*=90) reported using sublingual cannabis during the day and 29% (*N*=87) reported vaporizing cannabis during the day. **(B)** Of *N*=166 patients who responded to questions regarding nighttime use, 30% (*N*=50) reported sublingual use at night and 31% (*N*=52) reported vaporizing at night to manage pain while they slept. **(C)** Of *N*=99 patients who used inhalable cannabis, 23% (*N*=23) were unable to estimate the amount of THC in the inhalable product they used most often. The most commonly reported inhaled THC potency was 71% or more, which likely reflects a large proportion of patients using vaporizable cannabis oil. **(D)** Of *N*=126 patients who reported using ingestible cannabis products, 25% (*N*=32) were unable to estimate the amount of THC they take at one time. The most commonly reported oral THC dose was 1 mg or less at a time. Color images are available online.

At their first follow-up, 29% (*N*=90) reported using sublingual cannabis during the day and 30% (*N*=50) reported sublingual use at night (Fig. 2A, B). Twenty-nine percent of respondents (*N*=87) reported vaporizing cannabis during the day, and 31% (*N*=52) reported vaporizing at night to manage pain while they slept (Fig. 2A, B).

When asked about cannabinoid content, 99 patients reported using inhalable cannabis. When asked how much THC is in the inhalable cannabis product they use most often, 23% (*N*=23) were unable to make an estimate (Fig. 2C). The most commonly reported inhaled THC potency was 71% or more (vaporized oil). One hundred twenty-six patients reported using ingestible cannabis products, and when asked how much THC they use at a single time, 25% (*N*=32) were unable to make an estimate (Fig. 2D). The most commonly reported oral THC dose was 1 mg or less at a time.

### Primary outcome

**FIG. 3.**
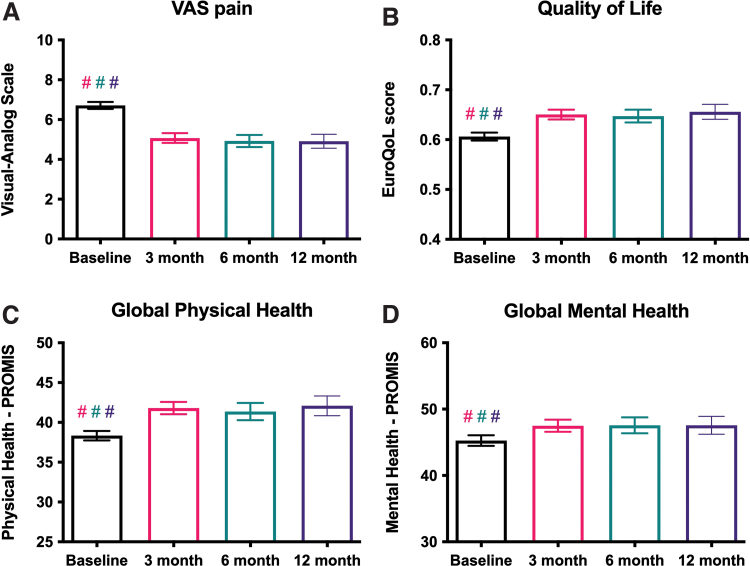
Primary outcome: analgesic efficacy of medical cannabis use. **(A)** Pain scores, as measured on the VAS, were significantly improved at ∼3 months (56 days, *N*=385 patients), ∼6 months (140 days, *N*=226 patients), and ∼12 months (308 days, *N*=157 patients) after the initiation of cannabis therapy, compared with baseline. **(B)** QoL at all follow-up time points was significantly improved compared with baseline, as measured by EuroQoL EQ-5D. **(C)** Global physical health at all follow-up time points was significantly improved compared with baseline, as measured by PROMIS. **(D)** Global mental health at all follow-up time points was significantly improved compared with baseline, as measured by PROMIS. For all panels, no follow-up time points were significantly different from any other follow-up time point. For all panels, # indicates *p*<0.0001, mixed-effects analysis with Tukey's multiple comparisons test, and bars represent mean with 95% confidence interval. PROMIS, Patient-Reported Outcomes Measurement Information System; VAS, Visual Analog Scale. Color images are available online.

VAS pain was significantly reduced at 3, 6, and 12 months after the initiation of cannabis therapy (6.7 vs. 5.2 at first follow-up, *N*=385, *p*<0.001, Fig. 3A). QoL (0.61 vs. 0.65 at first follow-up, *N*=373 *p*<0.001), global physical health (GPH, 38.3 vs. 41.4 at first follow-up, *N*=373, *p*<0.001), and global mental health (GMH, 45.4 vs. 47.2 at first follow-up, *N*=373, *p*<0.001) were all significantly improved at 3, 6, and 12 months after the initiation of cannabis therapy (Fig. 3B–D). There were no significant differences in VAS, QoL, GPH, or GMH scores between the 3-, 6-, and 12-month follow-ups (Fig. 3A–D).

### Secondary outcomes

**FIG. 4.**
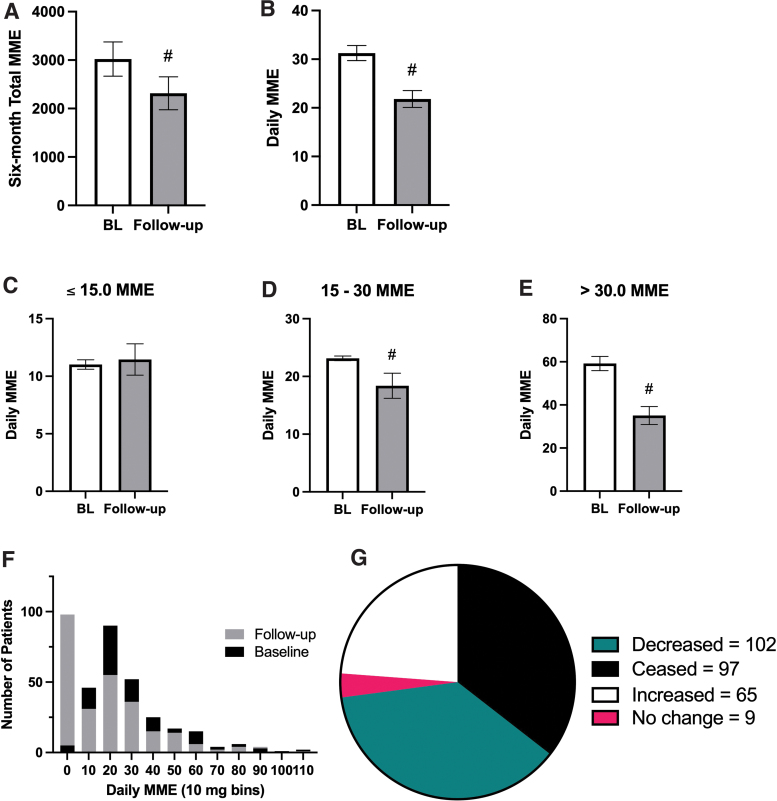
Secondary outcome: impacts on prescription opioid consumption after initiating medical cannabis use. **(A)** Six-month total opioid prescriptions (in morphine milligram equivalent, MME) are significantly lower after the initiation of cannabis therapy (gray bars, *p*<0.0001). **(B)** Normalized daily opioid doses (in MME) are significantly lower after the initiation of cannabis therapy (gray bars, *p*<0.0001). **(C)** For patients who were on low doses of opioids before cannabis certification (*N*=67, ≤15 MME/day), cannabis therapy did not significantly impact their daily opioid prescription (*p*>0.05). **(D)** For patients who were on moderate doses of opioids (*N*=122, 15–30 MME/day), cannabis initiation was associated with a 20.6% decrease in daily opioid prescription (*p*<0.0001). **(E)** For patients who were on high doses of opioids (*N*=83, >30.0 MME/day), cannabis initiation was associated with a 40.7% decrease in daily opioid prescription (*p*<0.0001). **(F)** Superimposed histogram of daily MME before (baseline, black bars) and after (follow-up, gray bars) cannabis therapy initiation, binned by 10 MME/day. 0–9 MME bin (far left) includes 97 patients who ceased opioids, represented as 0.00 MME/day. X axis clipped at 119 MME/day, excluding 7 patients from graphed data. **(G)** Pie chart representation of all patients' opioid prescriptions after cannabis therapy initiation: 37.4% decreased (teal), 35.5% ceased (black), 23.8% increased opioid use (white), and 3.2% remained stable (pink). For **(A, B, D)** # indicates *p*<0.0001 and bars represent mean with SEM. For **(A–D)**, Wilcoxon matched-pairs test was used. MME, morphine milligram equivalent; SEM, standard error of the mean. Color images are available online.

PDMP data were available for a subset of the total patients (*N*=358). Before the initiation of cannabis therapy, the 6-month total opioid use was an average of 3021 MME, with a median of 950 MME (95% confidence interval [CI]: 2324–3718). In the 6-month period following the initiation of cannabis therapy, the mean 6-month total opioid use fell to 2314 MME, with a median of 315.0 MME (95% CI: 1645–2984). Thus, the initiation of cannabis therapy was associated with a 23.4% reduction in the 6-month total opioid prescription (*p*<0.0001, Fig. 4A). Before the initiation of cannabis therapy, normalized daily opioid prescription was an average of 31.26 MME, with a median of 23.57 MME (95% CI: 28.19–34.33). In the 6-month period following the initiation of cannabis therapy, mean daily opioid prescription fell to 21.82 MME, with a median of 15.00 MME (95% CI: 18.41–25.23). Thus, the initiation of cannabis therapy was associated with a 30.2% reduction in daily opioid doses (*p*<0.0001, Fig. 4B).

Based on the commonly prescribed daily opioid dose of 15 MME/day, patients were split into high-opioid (>30.0 MME/day before cannabis therapy), moderate-opioid (15.0–30.0 MME/day), and low-opioid groups (≤15.0 daily MME). Patients in the low-opioid group did not exhibit any differences in daily MME prescriptions before and after cannabis initiation (11.02 MME/day vs. 11.45, *p*=0.96, Fig. 4C). Patients in the moderate-opioid group experienced a 20.6% reduction in mean daily MME (23.14 MME/day vs. 18.38, *p*<0.0001, Fig. 4D). Patients in the high-opioid group experienced a 40.7% reduction in mean daily MME (59.2 MME/day vs. 35.09, *p*<0.0001, Fig. 4E). In the 6-month period before cannabis certification, the most commonly prescribed daily opioid doses were between 15 and 29 MME (Fig. 4F). However, after cannabis certification, the most common daily dose was 0–9 MME (*N*=98). In fact, after initiating cannabis therapy, 97 of these 98 patients (35.4% of all patients) were able to cease using opioids entirely and an additional 37.2% (*N*=102) were able to reduce their opioid doses (Fig. 4G). Thus, 72.6% of the patients in this study were either able to cease or decrease their opioid consumption.

**FIG. 5.**
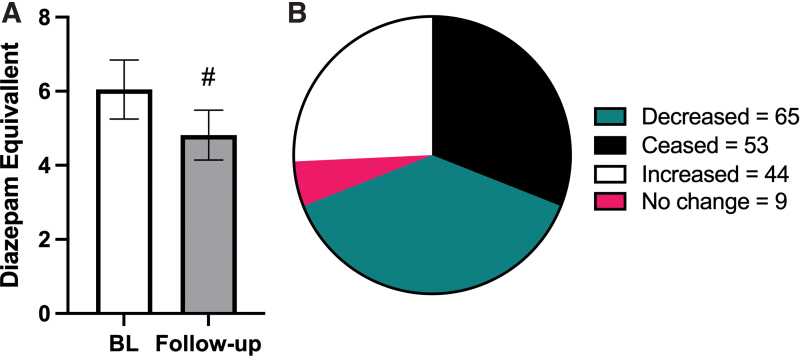
Secondary outcome: impacts on prescription benzodiazepine consumption after initiating medical cannabis use. **(A)** Daily diazepam milligram equivalent doses are significantly lower at follow-up, compared with the 6-month period before cannabis initiation. **(B)** Of *N*=171 patients, 31% were able to cease using benzodiazepines entirely (black), and an additional 38% (teal) were able to reduce their benzodiazepine doses compared with baseline. For **(A)** # indicates *p*<0.0001 and bars represent mean with SEM, Wilcoxon matched-pairs test. Color images are available online.

Population studies have shown that when a state enacts medical cannabis legislation, prescription drug costs for benzodiazepines also decrease.^18^ Upon analyses of the PDMP data, we observed that patients taking benzodiazepines before initiating cannabis therapy experienced a 20% reduction in daily diazepam mg equivalent doses (6.05 vs. 4.82, *p*<0.0001, Fig. 5A). Thirty-one percent of all patients were able to cease using benzodiazepines entirely (*N*=53), and an additional 38% (*N*=65) were able to reduce their benzodiazepine doses compared with baseline (Fig. 5B); thus, 69% of the patients in this study were either able to cease or decrease their benzodiazepine consumption.

## Discussion

Literature regarding the effectiveness of cannabis for chronic pain conditions is mixed. In a recent literature review, only 5 of 13 studies reported a significant treatment effect of cannabis.^23^ However, the authors concluded that cannabis may have a modest analgesic effect for chronic pain given that VAS pain intensity scores were decreased in multiple studies.^23–27^ Our findings not only indicate improvements on the subjective VAS, they also suggest functional improvements given the changes observed in QoL, GMH, and GPH scores. These data support the analgesic effect of cannabis for orthopedic pain, although the possibility of selection bias may limit the generalization of these findings.

Our data are in alignment with several previous studies, demonstrating that cannabis initiation is associated with decreased opioid use in patients with chronic pain.^13–15,28^ To our knowledge, our study is the first to identify subpopulations of opioid-consuming patients, most likely to cease or diminish their opioid consumption. Patients consuming greater than 30.0 MME/day experienced the greatest magnitude of opioid reduction. However, the most common baseline opioid prescriptions were in the 15–30 MME group. Thus, patients with high opioid doses may experience the greatest degree of dose reduction, but those in the 15–30 MME/day group may be the largest population to benefit from the opioid-sparing effects of cannabis. These results are highly encouraging because those at the highest risk for dangerous and unwanted opioid side effects appear to benefit the most from the opioid-sparing effects of cannabis.

Despite their controversial utility for pain management, benzodiazepines are commonly prescribed to chronic pain patients.^29^ Epidemiological analysis suggests that patients use cannabis as an adjunct or alternative to benzodiazepines.^30^ Our prospective study found that 69% of patients either cease or decrease benzodiazepine use. Compared with patients with opioid prescriptions, overdose mortality is 10-fold higher in patients who are coprescribed benzodiazepines and opioids.^31^ Thus, cannabis may play a role in decreasing the mortality of combined drug overdoses.

Mood disorders such as major depression and anxiety are extremely common in chronic pain patients, and pre-clinical studies indicate a neurobiological link between somatic pain and negative affect.^32–36^ Our data support the utility of cannabis for comorbid affective disorder in pain patients, given the improvements we observed in GMH. Previous studies have shown that CBD has anxiolytic properties in both animals and humans and reduces drug craving in individuals using illicit opioids.^37–39^ THC also appears to be critically involved in the therapeutic benefits of cannabis. In humans, whole cannabis flower and higher levels of THC have recently been linked with greater analgesic efficacy.^40^ These results suggest that cannabis products containing both CBD and THC have the potential to simultaneously relieve the affective and somatic components of pain, while supporting negative affect and diminishing the risk of drug overdose.

Sublingual cannabis and vaporized cannabis were the most common methods of administration, differing from previous studies where smoking was more prevalent.^41^ These discrepancies are likely attributable to PA's regulatory structure, which at the time of data collection did not permit smokable inflorescence. A large proportion of patients were unable to estimate the cannabinoid content of their most frequently used product, highlighting the limitations of observational studies and the necessity of controlled trials. Although there are several clinically validated measures for characterizing cannabis use patterns, other inventories were largely designed to detect the presence and magnitude of problematic drug use or cannabis use disorder (CUD).^42,43^ Most currently available tools lack medically relevant questions, and they tend to regard intoxication as a risk factor for CUD rather than a medication side effect. There is a critical need for validated inventories that capture the phenotypes and use patterns of cannabis.^44^

Because cannabis is not covered by insurance, it is possible that our findings may have been biased by the disproportionate inclusion of people with higher incomes. However, the individually titrated serving size, total amount of cannabis consumed, and thus the cost for a 30-day supply of medical cannabis are both difficult to calculate and highly variable. At the time of publication, the average price for a 500 mg vaporizer cartridge was $51, and the average bottle of tincture was $56.^45^ Most participants in this study used cannabis two to three times per day, which would roughly equate to a monthly cost of $40–90 for vaporizer cartridges, and $56–168 for sublingual tincture. These estimates are somewhat in agreement with the cannabis industry point-of-sale data, which suggest that at the time of the study, the average cannabis purchase in PA was approximately $112.^45^

THC's psychotropic side effects present a unique clinical challenge. Similar to opioids, THC is a rewarding substance that produces tolerance, dependence, and withdrawal.^46^ In the current study, the degree of education and guidance provided by the physician at the time of medical cannabis certification may have contributed to the low prevalence of self-reported intoxication. However, widespread and reproducible management of impairment and other side effects would be greatly enhanced by direct, in-depth physician management of cannabis therapy. Undoubtedly, this depth of care would require a substantial expansion of cannabis education for doctors, given that only 15–40% of trainees receive formal cannabis education during medical school.^47,48^

This study has several limitations. Participants self-enrolled, thus introducing the possibility of a selection bias. Because we relied on PDMP data, a key assumption is that patients consumed opioids as prescribed. There is the possibility that patients did not finish their doses or they were diverted. We were also unable to identify which cannabis products, at which cannabinoid potencies, delivered by which administration route are the most efficacious for orthopedic pain. Similarly, we were unable to define a dose or frequency regimen that may have provided the greatest therapeutic efficacy. Due to the observational nature of the study, we were also unable to estimate the magnitude of the placebo effect on our observed outcomes.

## Conclusions

Our results support the use of cannabis as an effective analgesic and prescription drug-sparing therapy. In patients with chronic musculoskeletal noncancer orthopedic pain, cannabis reduces pain, improves mental and physical health, and improves QoL. These improvements occur within 3 months of regular cannabis use and appear to plateau thereafter. Our results show an objective association between the initiation of cannabis therapy and the reduction of both opioid and benzodiazepine prescriptions. Interestingly, therapeutic benefits occur with infrequent-to-moderate cannabis use, and the majority of patients appear to self-titrate in a manner that produces beneficial effects in the absence of intoxication. However, the majority of patients are unable to estimate the cannabinoid content of their most frequently used products. Thus, placebo-controlled trials are necessary to definitively associate cannabinoid content and route of administration with analgesic efficacy, medication sparing, and other outcomes.

## Supplementary Material

Supplemental data

## Abbreviations Used

CBD: cannabidiol
CI: confidence interval
COVID-19: coronavirus disease 2019
CUD: cannabis use disorder
GMH: global mental health
GPH: global physical health
iMCU: Inventory of Medical Cannabis Use
MME: morphine milligram equivalents
PDMP: Pennsylvania Drug Monitoring Program
PROMIS: Patient-Reported Outcomes Measurement Information System
QoL: quality of life
ROCDR: Rothman Orthopaedic Cannabis Data Repository
SEM: standard error of the mean
THC: tetrahydrocannabinol
VAS: Visual Analog Scale
