# Supplementary material for: Perceived Efficacy, Reduced Prescription Drug Use, and Minimal Side Effects of Cannabis in Patients with Chronic Orthopedic Pain
Source: Cannabis Cannabinoid Res. 2022 Dec 5;7(6):865–75. doi: 10.1089/can.2021.0088 (PMC9784606; doi:10.1089/can.2021.0088)
Supplement: Supplemental data [file Suppl_Data.zip › RO_Cannabis_Patient_Handout_3-8-21.pdf]

Understanding cannabis: cannabidiol (CBD) & tetrahydrocannabinol (THC):
The main chemical compounds (cannabinoids) in cannabis are CBD and THC. They have unique mechanisms of action, but are thought to be most effective when used together.

- THC can be intoxicating (can make you feel "high" and may have additional side effects), particularly at dosages of five or more milligrams.
- CBD is non-intoxicating and can help reduce inflammation and improve sleep. CBD is very well tolerated even at high doses.

The CBD:THC ratio and the total amount of THC will affect the intoxication level. The higher the ratio of CBD to THC, the less likely you are to experience THC's common side effects. The lower the ratio, the greater potential for intoxication.

- 10:1 – 10x the amount of CBD to THC (less intoxicating, better for daytime usage)
- 1:1 – may be more intoxicating (better for nighttime dosage as it may be more sedating)

Effects of various strains:
Many medical cannabis products come from specific plant strains that have different effects on individuals. Some may be sedating, some may be energizing , some may cause muscle relaxation, euphoria, or anxiety. It's believed that these different effects are not only related to the amount of CBD and THC in these plants, but also aromatic compounds known as terpenes. It's important to track which cannabis strains (and even more importantly their ratio of THC to CBD) are most effective for you and to continue using similar products over time.

Delivery method options:

- ☐ Vaporization ("vaping"): inhaling vapor – not smoke – from cannabis oil or plant material that is heated to a temperature below the point of combustion. Heat allows cannabinoids and other molecules to detach from plant matter and become active, without exposing your lungs to smoke. It will take effect within 15 minutes and can last 2-3 hours. When starting out is a good idea to take a single inhalation and wait 15 to 20 minutes to assess how you feel before taking another single inhalation.

  1:1 CBD THC strains, Flower THC < 20%
  Oil THC <70%

- ☐ Pill: Can take 45 minutes to 2 hours to be effective, but typically last the longest (4-6 hours).

- ☐ Sublingual tinctures: drops absorbed through blood vessels under the tongue, can take 15-30 minutes to take effect and are a good way to begin using cannabis if you are not used to inhalation methods, or prefer not to vape.

  Initial oral THC dose of 2-3 mg combined with CBD. May increase by 1-2 mg THC as needed.

- ☐ Topical: ointment that can be rubbed directly onto the skin on an affected area. Patients report the best effects occur from rubbing these products completely into the skin several times a day as needed. Topical products carry very little risk for intoxication.

  Topical THC for localized pain.

How to minimize intoxication from THC:
It's important to read all product labels and speak to the dispensary staff about how to start with low dosages of THC combined with CBD. It's recommended that patients start with a low dose in the evening and monitor for any side effects. Your body may develop a tolerance to THC with daily use. After a few nights you can increase the dose as needed by no more than a few milligrams of THC at a time and start using an appropriate dosage during the day as needed.

Strategies for how to minimize intoxication:
1.) Vaporization: Strains with low percentage THC and equal or higher percentage CBD.
2.) Pill or tincture: Low dose THC (1 to 5 mg) combined with higher dose CBD (5 or more mg).
3.) Topical: THC products do not typically cause intoxication.

Additional resources on medical cannabis can be found on Projectcbd.org and Jefferson's Lambert Center resource page: www.jefferson.edu/lambertcenter.
